# Associations of Mediterranean Diet and *a Posteriori* Derived Dietary Patterns with Breast and Lung Cancer Risk: A Case-Control Study

**DOI:** 10.3390/nu10040470

**Published:** 2018-04-11

**Authors:** Beata Krusinska, Iwona Hawrysz, Lidia Wadolowska, Malgorzata Anna Slowinska, Maciej Biernacki, Anna Czerwinska, Janusz Jacek Golota

**Affiliations:** 1 Department of Human Nutrition, University of Warmia and Mazury in Olsztyn, Sloneczna 45f, 10-718 Olsztyn, Poland; iwona.hawrysz@uwm.edu.pl (I.H.); lidia.wadolowska@uwm.edu.pl (L.W.); malgorzata.slowinska@uwm.edu.pl (M.A.S.); 2 Department of Surgery, University of Warmia and Mazury in Olsztyn, 11-041 Olsztyn, Poland; maciej.biernacki@uwm.edu.pl; 3 Independent Public Complex of Tuberculosis and Lung Diseases in Olsztyn, 10-357 Olsztyn, Poland; aczerwinska@pulmonologia.olsztyn.pl; 4 Clinic of Thoracic Surgery, Medical Center Ars Medica, 10-513 Olsztyn, Poland; januszgolota@vp.pl

**Keywords:** breast cancer, lung cancer, dietary pattern, Mediterranean diet, adults

## Abstract

Lung cancer in men and breast cancer in women are the most commonly diagnosed cancers in Poland and worldwide. Results of studies involving dietary patterns (DPs) and breast or lung cancer risk in European countries outside the Mediterranean Sea region are limited and inconclusive. This study aimed to develop a ‘Polish-adapted Mediterranean Diet’ (‘Polish-aMED’) score, and then study the associations between the ‘Polish-aMED’ score and *a posteriori*-derived dietary patterns with breast or lung cancer risk in adult Poles. This pooled analysis of two case-control studies involved 560 subjects (280 men, 280 women) aged 40–75 years from Northeastern Poland. Diagnoses of breast cancer in 140 women and lung cancer in 140 men were found. The food frequency consumption of 21 selected food groups was collected using a 62-item Food Frequency Questionnaire (FFQ)-6. The ‘Polish-adapted Mediterranean Diet’ score which included eight items—vegetables, fruit, whole grain, fish, legumes, nuts and seeds—as well as the ratio of vegetable oils to animal fat and red and processed meat was developed (range: 0–8 points). Three DPs were identified in a Principal Component Analysis: ‘Prudent’, ‘Non-healthy’, ‘Dressings and sweetened-low-fat dairy’. In a multiple logistic regression analysis, two models were created: crude, and adjusted for age, sex, type of cancer, Body Mass Index (BMI), socioeconomic status (SES) index, overall physical activity, smoking status and alcohol abuse. The risk of breast or lung cancer was lower in the average (3–5 points) and high (6–8 points) levels of the ‘Polish-aMED’ score compared to the low (0–2 points) level by 51% (odds ratio (OR): 0.49; 95% confidence interval (Cl): 0.30–0.80; *p* < 0.01; adjusted) and 63% (OR: 0.37; 95% Cl: 0.21–0.64; *p* < 0.001; adjusted), respectively. In the middle and upper tertiles compared to the bottom tertile of the ‘Prudent’ DP, the risk of cancer was lower by 38–43% (crude) but was not significant after adjustment for confounders. In the upper compared to the bottom tertile of the ‘Non-healthy’ DP, the risk of cancer was higher by 65% (OR: 1.65; 95% Cl: 1.05–2.59; *p* < 0.05; adjusted). In conclusion, the Polish adaptation of the Mediterranean diet could be considered for adults living in non-Mediterranean countries for the prevention of the breast or lung cancers. Future studies should explore the role of a traditional Mediterranean diet fitted to local dietary patterns of non-Mediterranean Europeans in cancer prevention.

## 1. Introduction

Based on statistics from the GLOBOCAN [1], the number of cancer cases is growing rapidly worldwide, and in 2012, the number of cases increased to 14 million, including 8 million deaths. Lung cancer in men and breast cancer in women are the most commonly diagnosed cancers, including in developed and developing countries [2]. Worldwide, in 2012, lung cancer accounted for about 17% of the total cancer cases and 24% of cancer deaths in males, and breast cancer accounted for about 25% of the total cancer cases and 15% of cancer deaths in females [2]. 

In Poland, the number of cancer cases has more than doubled over the last three decades and in 2013, was about 156 thousand, including 95 thousand deaths [3]. Lung and other respiratory cancers (trachea, bronchus) comprise about 19% of total cancer cases and 31% of cancer deaths in Polish males and breast cancer comprises about 22% of total cancer cases and 14% of cancer deaths in Polish females [3,4]. 

Cancer aetiology is multi-factorial and includes some predictors which cannot be modified, such as age, genetic predisposition and some environmental factors, whereas lifestyle factors, including smoking, physical activity and diet, are modifiable and can be changed [2,5]. Although there are many studies regarding the association of dietary factors with breast and lung cancer, the results remain inconsistent [6]. According to the World Cancer Research Fund [6,7], convincing evidence has only been obtained for ethanol and beta-carotene supplements for smokers as factors increasing the risk of breast and lung cancers, respectively. The consumption of fruits and foods containing carotenoids probably decreases the risk of lung cancer [6]. There is limited evidence suggesting that non-starchy vegetables, foods containing selenium and quercetin decrease the risk of lung cancer [6] (but red and processed meat, total fat, butter and retinol supplements (for smokers only) increase the risk). Evidence for the impact of the other food groups or nutrient intakes on the risk of breast and lung cancer is limited, and no conclusions have been drawn [6,7]. It is important to identify dietary factors which might be useful in cancer prevention. The similar epigenetic mechanisms of breast and lung cancer indicate common dietary causes [8]. 

An approach for assessing associations with cancer that is focused on single foods or nutrients is not sufficient due to diet complexity [6,9]. An alternative approach is to focus on overall dietary patterns (DPs) which express many different aspects of the diet [9]. Although there are a number of studies related to the dietary patterns and breast or lung cancer risk, the findings are inconclusive [10,11,12,13,14,15]. There is a need to use multiple methods to study dietary patterns and their association with cancer to obtain a complete picture, but this approach has not been used often [16,17,18]. The most common techniques used for dietary pattern identification are *a posteriori* analyses, mainly the Principal Component Analysis (PCA), which is based on observed correlations among dietary variables [11,12,19,20]. Less commonly used are *a priori* approaches based on previous knowledge regarding the health effects of dietary constituents and predefined diet quality scores, like the Mediterranean diet score or the Healthy Eating Index [21,22,23,24,25,26].

The traditional Mediterranean diet is characterized by high consumption of vegetables, legumes, fruits, fish, nuts, wholegrains including non-refined cereals, and olive oil (which is rich in mono-unsaturated fatty acids), low-to-moderate consumption of minimally processed dairy products, regular but moderate intake of wine during meals, low consumption of red meat, poultry, highly processed and energy-dense foods rich in saturated fatty acids and sugar [27]. The Mediterranean diet is a plant-based, well-balanced diet with beneficial health effects and in 2010, was included on the list of the intangible cultural heritage of humanity of United Nations Educational, Scientific and Cultural Organization (UNESCO) [28]. It is a dietary pattern typical of the Mediterranean regions in the early 1960s, such as Crete and other parts of Greece, Spain, Southern Italy and France [27]. The protective role of the Mediterranean diet in the prevention of non-communicable diseases has been well established [29]. There is considerable evidence that high adherence to the Mediterranean diet reduces the risk of several cancers [30]. However, its association with breast or lung cancer risk remains unclear [21,22]. To the best of the current authors’ knowledge, no previous study has assessed the association between adherence to the Mediterranean diet and breast or lung cancer risk in adults from Central or Eastern European countries, including Poland.

In the current study, it was hypothesized that the Polish adaptation of the Mediterranean diet could be considered for adults living in non-Mediterranean countries for the prevention of breast or lung cancer. Thus, the aim of the study was twofold: (1) to develop the Mediterranean diet score adapted to the Polish diet (‘Polish-adapted Mediterranean Diet’ score), and (2) to study associations between the ‘Polish-adapted Mediterranean Diet’ score and *a posteriori*-derived dietary patterns with breast or lung cancer risk in adult Poles.

## 2. Materials and Methods

### 2.1. Ethical Considerations

The study was approved by the Bioethics Committee of the Faculty of Medical Sciences, University of Warmia and Mazury in Olsztyn on 2 October 2013 (resolution no. 29/2013). All subjects gave their written informed consent for inclusion before they participated in the study.

### 2.2. Study Design and Sample Characteristics

**Figure 1:**
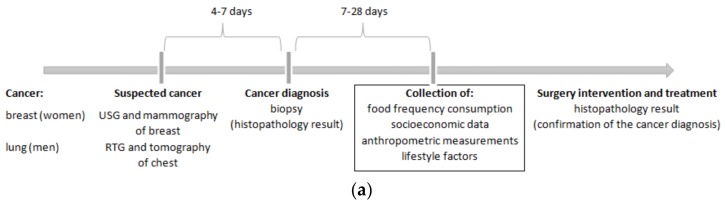

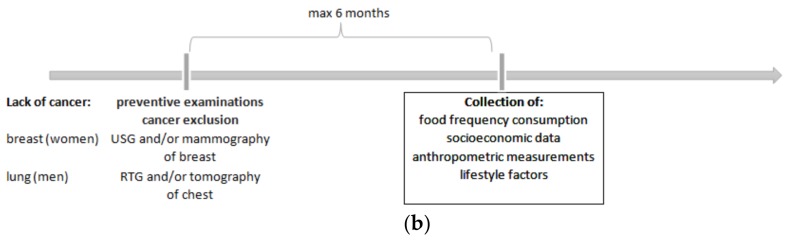
Time schemes of study design for (**a**) the cancer sample and (**b**) the control sample. USG—ultrasonography; RTG—digital X-ray examination.

Two separate study protocols with case-control designs were developed. These studies were conducted in 2013–2016 among adults from Northeastern Poland. The cancer sample involved women with breast cancer and men with lung cancer, diagnosed from biopsy histopathology results. All cancer cases were newly-diagnosed (primary diagnosis). The time from cancer diagnosis until participation in the study ranged from 7 days to 28 days (18 days on average) (Figure 1a). Cases of secondary cancer diagnosis as a recurrence of the disease or outcome of another cancer, or those with benign changes or other types of cancer, either at present or in the past were excluded. Individuals who were currently having, or who had previously had active treatment (e.g., chemotherapy, radiotherapy, hormonal treatment) also did not qualify for the study in order to avoid changes in dietary habits or other behaviours. Surgery intervention and decisions about treatment were made after the study (Figure 1a). 

The control sample were women who received a negative result from ultrasonography (USG) and/or mammography (MM) of the breasts and men who received a negative result from digital X-ray examination (RTG) and/or a computed tomography (CT) of the chest. The time since the cancer exclusion until participation in the study did not exceed 6 months (Figure 1b). Control subjects did not have any clinical symptoms or suspicion of any type of cancer in their medical history.

**Figure 2:**
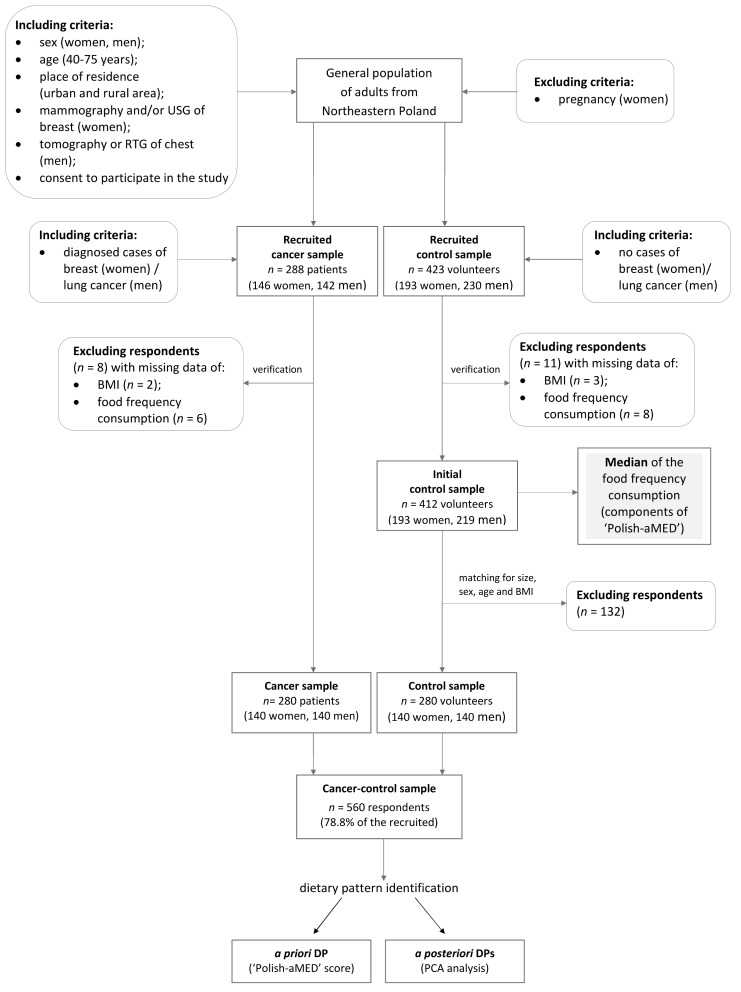
Flow chart of sample collection and study design. DP—dietary pattern; ‘Polish-aMED’—‘Polish-adapted Mediterranean Diet’; PCA—Principal Component Analysis; BMI—Body Mass Index; USG—ultrasonography; RTG—digital X-ray examination.

The cancer and control samples were chosen through a convenient and non-random selection. Breast cancer cases were patients diagnosed in the surgical oncology ward at the Hospital Ministry of Internal Affairs with Warmia and Mazury Oncology Centre in Olsztyn. Lung cancer cases were patients from pulmonary and oncology hospital wards in the Independent Public Complex of Tuberculosis and Lung Diseases in Olsztyn. The control sample were women and men who attended national screening programs for the early diagnosis of breast and lung cancer, respectively. The control sample was matched by size, age, sex and Body Mass Index (BMI) with cases. Details of the sample collection and study designs are shown in Figure 2. In total, the cancer and control samples involved 560 subjects, aged 40–75 (60.9 ± 7.2) years. The baseline characteristics of the cancer and control samples are presented in the results section.

### 2.3. Food Frequency Consumption

Dietary data were collected using a validated 62-item Food Frequency Questionnaire (FFQ-6) developed by Wadolowska and Niedzwiedzka [31]. A validation procedure of the questionnaire was carried out by Niedzwiedzka, Kowalkowska and Wadolowska (data not published, paper in preparation). In brief, the internal compatibility of the FFQ-6 and its ability to identify dietary patterns was tested for 97 girls and young women aged 13–21 years from the Warmia and Mazury region of Poland. The questionnaire was completed twice (test and retest). Two dietary patterns, DP1 and DP2, were derived in the test using the Principal Component Analysis. In the retest, the repeatability of the food composition was good for DP1 and satisfactory for DP2. The Kappa Fleiss values for food items were from 0.32 to 0.72 (on average, 0.52). Kappa values were moderate (from 0.41 to 0.60) for 50 food items (81% of total food items) and good (from 0.61 to 0.80) for 10 food items (16% of total food items). The compatibility of subject classification (into the same food frequency category in the test and retest) was from 51% to 89% (on average, 68%) for food items and 57% for DP1 and 43% for DP2. Therefore, the internal compatibility of the FFQ-6 and its ability to identify dietary patterns among girls and young women was considered acceptable to good. The wide scope of application for the FFQ-6 was confirmed by its use in a pilot randomized controlled trial among paediatric coeliac disease patients on a gluten-free diet [32]. Due to the greater precision of responses in adults than in younger individuals, higher repeatability of the FFQ was predicted [33], which was also confirmed by its use in the study of a genetic-specific nutritional intervention involving adult patients with non-alcoholic fatty liver disease [34].

In the study, an interviewer-administered version of FFQ-6 was used. Respondents were asked about food frequency consumption (6 categories to choose from) within the last 12 months prior to involvement in the study. The frequency of consumption was recalculated and expressed as times/day as follows: ‘never or almost never’ = 0; ‘once a month or less’ = 0.025; ‘several times a month’ = 0.1; ‘several times a week’ = 0.571; ‘daily’ = 1; ‘several times a day’ = 2 times/day. Some of the food items were combined by summing their frequency consumption into 21 food groups (Table 1, Table S1).

### 2.4. Polish-Adapted Mediterranean Diet Score

The Mediterranean diet score (MED), described by Fung et al. [35], was modified for the present analysis to an adapted Polish version of the MED called the ‘Polish-adapted Mediterranean Diet’ (‘Polish-aMED’) score. In developing the ‘Polish-aMED’ score, alcohol was excluded due to being an established risk factor for breast cancer [2,6,7], and the score included the ratio of vegetable oils to animal fat instead of the ratio of monounsaturated to saturated fatty acids, based on qualitative data. Due to the relatively low consumption of olive oil-derived monounsaturated fatty acids in non-Mediterranean countries, the total consumption of vegetable oils was considered.

The ‘Polish-aMED’ score was developed using medians of the frequency of consumption (times/day) of eight dietary items, calculated from the initial control sample as reference cut-offs (Table 2 and Table 3). Points were assigned for the frequency of consumption above the median for seven food groups—vegetables, fruit, whole grains, fish, legumes, nuts and seeds—as well as the ratio of vegetables oils to animal fat. An extra point was added for the frequency of consumption of red and processed meats below the median intake (Table 2 and Table 3). The ‘Polish-aMED’ score was calculated as the sum of points and was expressed in a range from 0 to 8. Three levels of the ‘Polish-aMED’ score were created a priori: low (0–2 points), average (3–5 points) and high (6–8 points).

### 2.5. Confounders

The potential confounders have been described previously [36] and are included in Supplementary Tables S2 and S3. Briefly, the socioeconomic status (SES) index was calculated as the sum of the values assigned to the individual response categories for each of three single SES factors (Table S2). The SES index values were logarithmized, and the tertiles of the SES were then created to identify respondents as having low, average or high SES. The adjusted logistic regression model was also included with the following confounders: age (continuous, years), sex (categorical, man/woman), type of cancer (categorical, breast/lung cancer), BMI (continuous, kg/m^2^), overall physical activity (categorical, low/moderate/high), smoking status (categorical, non-smoker/smoker) and abuse of alcohol (categorical, no/yes; Tables S2 and S3). These potential confounders were selected *a priori* according to current knowledge regarding common and possible factors for breast and lung cancer risk [6,7].

### 2.6. Statistical Analysis

The frequency of consumption of 21 food groups (expressed as times/day) was standardized and included in the Principal Component Analysis (PCA) with varimax rotation [37]. To identify the number of PCA-derived patterns to retain, the eigenvalues (from the correlation matrix of the standardized variables) >1.0 of a criterion, the break-point identified in the scree plot of the eigenvalues and the total variance in the frequency of food consumption were considered [37]. Rotated factor loadings with an absolute value of 0.30 or more were considered to significantly contribute to each dietary pattern, and a higher loading indicated a stronger association between a food group and a dietary pattern, although the value for meaningful factor loading was arbitrary [17,38]. Dietary patterns were named according to the highest food group loadings for each of the factors. The association between the frequency consumption of 21 food groups and the ‘Polish-aMED’ score was evaluated using Pearson’s correlation coefficients. The tertile intervals were calculated for each of the PCA-derived DPs and the levels were created for ‘Polish-aMED’ score. The percentage distribution of breast or lung cancer cases was compared by tertiles or levels of DPs using Pearson Chi^2^ test with Yates’ correction as necessary. The associations of DPs with breast or lung cancer risk were verified by logistic regression analysis. The odds ratio (OR) and 95% confidence interval (95% CI) were calculated. The references (OR = 1.00) were the control sample and the bottom tertile or lowest level of each DP. Two models were created: crude and adjusted for the potential confounders mentioned above. The level of significance of the odds ratio was assessed with a Wald test [37]. The statistical analysis was performed using STATISTICA software (version 10.0 PL; StatSoft Inc., Tulsa, OK, USA; StatSoft, Krakow, Poland). A *p*-value < 0.05 was considered statistically significant.

## 3. Results

In comparison with the controls (non-cancer), more cases of breast or lung cancer were identified in individuals who came from a village, had a lower education level or lower socioeconomic status, were less physically active, including physical activity at work and in leisure time, or were smokers, including former smokers (Table 4).

### 3.1. Food Frequency Consumption and Dietary Patterns

A significantly positive correlation with the *a priori*-developed ‘Polish-aMED’ score was found for the frequency consumption of seven (out of eight) components—vegetables (*r* = 0.49), whole grain products (*r* = 0.47), fruits (*r* = 0.46), nuts and seeds (*r* = 0.44), fish (*r* = 0.34) and legumes (*r* = 0.33)—and a negative correlation for one of its components—red and processed meats (*r* = −0.18; Table 1). The frequency of consumption of food groups by level of the ‘Polish-aMED’ score is shown in Supplementary Table S1. 

Using an *a posteriori* approach, three main dietary patterns were identified, which explained 33% of the variation in the frequency of consumption of 21 food groups (Table 1). The ‘Prudent’ DP was positively loaded by the frequency of consumption of vegetables, fruits, milk, fermented milk drinks and curd cheese, whole grain products, fish, legumes, nuts and seeds, vegetable oils, eggs, fruit, vegetable or vegetable-fruit juices, cereals, cheese and white meat (loadings from 0.30 to 0.55; Table 1). The ‘Non-healthy’ DP was positively loaded by the frequency of consumption of refined grain products, sugar, honey and sweets, red and processed meats, potatoes, animal fats, sweetened beverages, energy drinks and cheese (loadings from 0.30 to 0.71) and was negatively loaded by the frequency of consumption of whole grain products (loading −0.42; Table 1). The ‘Dressings and sweetened-low-fat dairy’ DP was positively loaded by the frequency of consumption of other edible fats (margarine, mayonnaise, dressings), sweetened milk drinks and flavoured homogenized cheese (loadings from 0.39 to 0.81) and negatively loaded by the frequency of consumption of animal fats (loading −0.65; Table 1). 

### 3.2. Dietary Patterns and Breast or Lung Cancer Risk

In the high level of the ‘Polish-aMED’ score, the number of breast or lung cancer cases was lower than in the low level by 25.6% points (40.8% vs. 66.4%; Table 5). In the upper tertile of the ‘Prudent’ DP, the number of cancer cases was a lower than in the bottom tertile by 11.8% points (46.8% vs. 58.6%; Table 5). In the upper tertile of the ‘Non-healthy’ DP, the number of cancer cases was higher than in the bottom tertile by 15.8% points (59.1% vs. 43.3%; Table 5). There were no significant differences in the number of cancer cases within the tertiles of the ‘Dressings and sweetened-low-fat dairy’ DP. These associations were confirmed in a logistic regression analysis (Table 5) with one exception in the adjusted model.

In the average (3–5 points) and high (6–8 points) levels of the ‘Polish-aMED’ score, the risk of breast or lung cancer was lower by 51% (OR: 0.49; 95% Cl: 0.30–0.80; *p* < 0.01; adjusted model) and 63% (OR: 0.37; 95% Cl: 0.21–0.64; *p* < 0.001; adjusted model), respectively, when compared to the low level (0–2 points) as a reference (Table 5). In the middle and upper tertiles of the ‘Prudent’ DP, the risk of cancers was lower by 43% (OR: 0.57; 95% Cl: 0.38–0.86; *p* < 0.01; crude model) and 38% (OR: 0.62; 95% Cl: 0.41–0.94; *p* < 0.05; crude model), respectively, when compared to the bottom tertile as a reference. This association was not significant after adjustment. In the upper tertile of the ‘Non-healthy’ DP, the risk of cancers was higher by 65% (OR: 1.65; 95% Cl: 1.05–2.59; *p* < 0.05; adjusted model) when compared to the bottom tertile as a reference. The ‘Dressings and sweetened-low-fat dairy’ DP was not significantly associated with the risk of breast or lung cancers (Table 5).

## 4. Discussion

In adults from Northeastern Poland, high or average adherence to the ‘Polish-aMED’ score significantly decreased the risk of breast or lung cancer, independently of confounders. The association between the ‘Prudent’ pattern and risk of cancer was weak and disappeared after adjustment. High adherence to the ‘Non-healthy’ pattern increased the risk of the breast or lung cancer. The ‘Dressings and sweetened-low-fat dairy’ DP was not significantly associated with the risk of either cancer.

High adherence to the ’Polish-aMED’ reduced the breast and lung cancer risk by 63%, and the average adherence to this pattern by 51%. This strong association was found even though Poland is a non-Mediterranean country and the dietary habits of the Polish population do not closely resemble the traditional Mediterranean diet. These results are consistent with previous outcomes from other countries which have found a beneficial effect of the Mediterranean pattern on breast and lung cancer risk [17,19,23,24,26,41,42]. High adherence to the Mediterranean pattern was associated with a 6% lower breast cancer risk in the European Prospective Investigation into Cancer and Nutrition (EPIC) study [23] and in an updated meta-analysis [30] and from 15% lower in a cohort study in France [19] to 44% lower in a Spanish case-control study [17]. The results of epidemiological research into the effect of a Mediterranean diet pattern on lung cancer varied depending on the smoking status. In large European and Australian studies, high adherence to the Mediterranean diet has been associated with lower scores on the dietary inflammatory index and a 15% reduced lung cancer risk among former smokers [41], a 62% reduced lung cancer risk among current smokers [26] and an 80–90% reduced risk among heavy smokers [24,42]. 

The beneficial effects of a Mediterranean diet on cancer prevention might be explained by a number of biological pathways. The Mediterranean diet is rich in various plant-based foods, such as fruits and vegetables, which provide many bioactive compounds (flavonoids, carotenoids, vitamin C, A, E, and folate) which can neutralize free radicals and reduce oxidative DNA damage [29]. Since the Mediterranean diet contains phytoestrogens that display oestrogen-like effects and may compete with oestrogens to bind to oestrogen receptors, it can decrease levels of endogenous oestrogens and decrease the risk of hormone-related breast cancer [29]. Furthermore, whole grains and vegetables high in fibre and with a low glycaemic index help with weight loss and reduce insulin-like growth factor and insulin resistance, which are both also related to cancer risk [43]. The Mediterranean diet’s favourable fatty acid profile, with a high monounsaturated fatty acids (MUFA) to saturated fatty acids (SFA) ratio and polyunsaturated fatty acids (PUFA) *n*-3 to PUFA *n*-6 ratio, is associated with an anti-inflammatory effect through inhibition of eicosanoids derived from arachidonic acid [44]. Thus, the beneficial effects of the Mediterranean diet are the result of biologic interactions between its different components.

An inverse association between the Mediterranean diet and cancer incidence has been reported in most studies, but not in all. In a Greek-Cypriot case-control study [21], a German cross-sectional study [25], and American [45], British [18,46], Dutch [47] and Swedish [22] cohort studies, no significant association was found between the Mediterranean diet and breast cancer risk. The discrepancy could be explained by different approaches in developing the Mediterranean pattern, e.g., selecting different dietary items (moderate wine drinking, meat or dairy consumption) [30], using various cut-offs, using different methods of statistical analysis and including different confounders. 

The current study found that the ‘Prudent’ pattern reduced the risk of breast and lung cancer by 38–43%. However, this beneficial effect disappeared after adjustment, so the association was weaker than for the ‘Polish-aMED’ pattern. Besides the ‘Polish-aMED’s components, the ‘Prudent’ DP also included juices, cereals, dairy, eggs and white meat. These dietary items contain both beneficial components and those considered to be unhealthy when eaten in greater amounts (e.g., mono- and disaccharides). Similar to the present study, no significant association was found between breast cancer risk and the ‘cereals/milk/dairy’ DP [21], ‘vegetable’ DP [12] and the ‘prudent’ DP which was rich in low-fat dairy, vegetables, fruits, whole grains and juices [17]. However, in many studies, ‘prudent’ DPs—rich in fruit, vegetables, low-fat dairy [48], fish, whole grains, juices [36] and poultry [10] or characterized by high consumption of fruits and vegetables only, such as the ‘plant-based’ [12], ‘fruit and salad’ [20], ‘salad vegetables’ [13] and ‘antioxidants’ DPs [14]—have been associated with a 15–56% lower risk of breast cancer [10,12,20], a 25–39% lower risk of lung cancer, including Caucasian never smokers [13,14,48] or a 52% lower risk of breast or lung cancer in a pooled analysis [36]. This protective effect probably results from the diet being high-quality and rich in bioactive compounds, including specific peptides, fatty acids, phenolics, vitamins, minerals and fibre. Conversely, in a North American study, the ‘prudent’ DP comprising low-fat dairy products, whole grains, vegetables, fruits, legumes and vegetable or fruit juices increased the risk of breast cancer by 1.42-fold [49]. This result is contrary to conventional wisdom and to the results of other studies. In the USA, the ‘prudent’ DP diet is relatively higher in carbohydrates and fat than the ‘prudent/healthy’ DPs in the diets of European countries. The differences in these associations could result from differences in the study design, population under study, secular trends in food supply or different definitions of the ‘prudent/healthy’ diet and characteristics of its foods [49]. 

In the present study, high adherence to the ‘Non-healthy’ DP increased the risk of breast or lung cancer by almost 2-fold and confirmed the findings related to the ‘processed & fast food’ DP in the authors’ previous study [36]. These findings are consistent with international data. Dietary patterns characterized by high intakes of processed meat, high-fat dairy, refined grains, sweets and caloric drinks, described as ‘Western’, ‘unhealthy’ [10,17,19] or ‘high-meat’ [15], ‘pork, processed meat and potatoes’ DPs [13] were associated with an increased risk of breast cancer—from 20% in a French cohort study [19] and approximately 1.5-fold in a Spanish case-control study [17]. They were also associated with a 2.6-fold increased risk of lung cancer in a Dutch cohort study [13] and an approximately 3-fold increased risk among Uruguayan men [15]. This negative effect probably resulted from a diet rich in foods with high energy density and a high glycaemic index, such as processed foods, because of their high fat and sugar content, which is related to elevated levels of energy balance and insulin resistance [43]. Moreover, a high consumption of red meat may also be associated with an almost 2-fold increased risk of lung cancer [24]. Red meat is a source of saturated fat, iron and some mutagenic compounds, including *N*-nitroso compounds, heterocyclic amines and polycyclic aromatic hydrocarbons, which have been related to cancer promotion through inflammatory effects as well as the generation of free radicals and promotion of oxidative stress [24]. However, in some studies, the ‘Western’ [11], Greek ‘meat/potatoes’ [21] and Californian ‘high-protein’ (meat, fried foods and fat) [12] diets were not significantly associated with breast cancer risk.

In the current study, no associations were found between the ‘Dressings and sweetened-low-fat dairy’ pattern and breast or lung cancer risk. This pattern included dairy with a potentially beneficial effect on health (even though it was sweetened), and dressings with higher fat content with potentially negative effects on health. This may have influenced the neutral character of the final results in regard to cancer incidence. In other studies, similar patterns were not selected, so a direct comparison of our results is impossible. 

To the authors’ best knowledge, this is the first Polish study regarding the association between Mediterranean patterns and breast or lung cancer risk, and the second study regarding *a posteriori*-derived dietary patterns in this area. Overall, the data highlight the beneficial effects of the ‘Polish-adapted Mediterranean Diet’ and the harmful effects of the ‘Non-healthy’ pattern on breast and lung cancer risk in adults from Northeastern Poland. These findings may be helpful for improving cancer prevention and making public dietary recommendations that are generalized and not focused on one type of cancer. Further studies in the Polish population as well as in other non-Mediterranean populations are needed to clarify the associations between diet and cancer risk.

There were some limitations in the current study, including the case-control design which is susceptible to recall and selection bias. The matching design in closely-related cases and controls often shows stronger diet–disease associations than other study designs [50]. However, matching by sample size, age, sex and BMI was needed to reduce variability due to background variables. Due to the multifactorial aetiology of cancer, a fully adjusted model for the diet–cancer association, including many potential confounders, was calculated. However, it was not possible to include all potential confounders. Thus, the possibility of residual confounding by factors that have not been evaluated cannot be ruled out. Another limitation is a lack of quantitative data regarding food and nutrient intake, although current evidence shows the limitations in concluding when single nutrient components are considered [9]. 

The strengths of the study are the identification of dietary patterns which represent the overall combination of usual consumed food and the consideration of the overall health effects (synergistic or opposed) of many single dietary items [9]. Secondly, two methods were used (*a priori* and *a posteriori*) to identify dietary patterns. By using both approaches for the same dataset, complementary outcomes were provided, which allowed us to broadly assess the respondents’ diets [38]. Thirdly, a validated interviewer-administrated FFQ was used with adequate-to-high internal repeatability [31] to collect dietary data and take measurements of body weight and height instead of using declared data. Finally, although a number of studies have explored the association between dietary patterns and cancer risk, none have reported results from adults from Central or Eastern Europe. The current paper fills this important gap by exploring the link between different dietary patterns, including the ‘Polish-adapted Mediterranean Diet’, and cancer risk in adults from Northeastern Poland. 

## 5. Conclusions

The present study provides interesting insight into the strong beneficial effects of high and average adherence to the ‘Polish-adapted Mediterranean Diet’, and the harmful effects of high adherence to a ‘Non-healthy’ dietary pattern on breast and lung cancer risk among adults from Northeastern Poland. The adaptation of the Mediterranean diet to the Polish diet was developed by including total oil consumption instead of just olive oil, and excluding alcohol. The study’s findings provide a good basis for recommending the Polish adaptation of the Mediterranean diet for adults living in non-Mediterranean countries for the prevention of breast and lung cancer. Furthermore, this study reinforces evidence that an unhealthy dietary pattern consisting of highly processed foods with a high content of sugar and animal fat, should be avoided to prevent cancer. 

Future studies should explore the role of the traditional Mediterranean diet fitted to local dietary patterns of non-Mediterranean Europeans, in real-life scenarios, to determine whether it could be a valuable lifestyle strategy for cancer prevention.

## Figures and Tables

**Table 1:** Factor loadings for food groups in Principal Component Analysis (PCA)-derived dietary patterns and the Pearson’s correlation coefficients for food groups in the ‘Polish-aMED’ (*n* = 560).

Food Groups	PCA-Derived Dietary Patterns			‘Polish-aMED’ Score
	‘Prudent’	‘Non-Healthy’	‘Dressings and Sweetened-Low-Fat Dairy’	
Vegetables	**0.55**	0.00	0.04	0.49 *
Fruits	**0.55**	0.02	−0.04	0.46 *
Milk, fermented milk drinks, curd cheese	**0.54**	0.00	0.28	0.28 *
Whole grain products	**0.53**	**−0.42**	−0.03	0.47 *
Fish	**0.51**	−0.05	0.05	0.34 *
Legumes	**0.48**	−0.01	−0.14	0.33 *
Nuts and seeds	**0.46**	−0.28	−0.16	0.44 *
Vegetable oils (including olive oil)	**0.44**	0.27	−0.04	0.30 *
Eggs	**0.43**	0.24	−0.01	0.18 *
Fruit, vegetable, vegetable-fruit juices	**0.39**	0.19	0.06	0.12 *
Cereals	**0.35**	−0.07	0.19	0.18 *
Cheese	**0.31**	**0.30**	0.08	0.11 *
White meat	**0.30**	0.28	0.22	0.11 *
Refined grain products	−0.22	**0.71**	0.12	−0.32 *
Sugar, honey and sweets	−0.02	**0.60**	0.09	−0.10 *
Red and processed meats	0.11	**0.56**	0.04	−0.18 *
Potatoes	0.03	**0.52**	0.04	−0.10 *
Animal fats	0.12	**0.47**	**−0.65**	−0.16 *
Sweetened beverages, energy drinks	0.03	**0.35**	−0.13	−0.02
Other edible fats (margarine, mayonnaise, dressings)	−0.06	0.17	**0.81**	−0.01
Sweetened milk drinks and flavoured homogenized cheese	0.28	0.22	**0.39**	0.05
Ratio of vegetable oils to animal fat	NA	NA	NA	**0.37**
Share in explaining the variance (%)	14	12	7	NA

‘Polish-aMED’—‘Polish-adapted Mediterranean Diet’ (range of points: 0–8); NA—not applied; bolded values are marked for the main components of PCA-derived dietary patterns with absolute loadings ≥0.3 and for eight components of the ‘Polish-aMED’ score; * *p* < 0.05, test of significance for Pearson’s correlation coefficients.

**Table 2:** Description of food groups for the ‘Polish-adapted Mediterranean Diet’ score (0–8 points) calculation.

Food Group/Dietary Items	Criteria for 1 Point
Vegetables: raw or cooked: cabbage, brussels sprouts, cauliflower, broccoli, kale, carrot, pepper, spinach, endive, lettuce, leek, celery, parsley, tomato, cucumber, cabbage, zucchini, pumpkin, eggplant, beets, parsnips, onion, garlic, radish, turnip, artichoke, asparagus, salads with mixed vegetables	Greater than median intake (times/day) *
Fruit: apricots, cherries, nectarines, peaches, plums, grapes, kiwis, oranges, mandarins, grapefruit, lemons, pomelos, pineapple, watermelon, melon, fresh dactyls, fresh figs, strawberries, raspberries, blackberries, blueberries, currants, bananas, apples, pears, avocado	Greater than median intake (times/day) *
Whole grains: whole-grain bread, whole-grain groats, brown rice, wholemeal pasta	Greater than median intake (times/day) *
Fish: freshwater fish (perch, panga, trout, carp, eel,) and marine fish (cod, salmon, sardines, hake, herring, tuna, mackerel, halibut)	Greater than median intake (times/day) *
Legumes: fresh or canned: corn, green beans, dry seeds of legumes in dishes: beans, soybeans, peas, chickpeas, hummus	Greater than median intake (times/day) *
Nuts and seeds: peanuts, hazelnuts, walnuts, almonds, pistachios, cashews, coconut, chestnuts,pumpkin seeds, sesame seeds, sunflower seeds, wheat germ	Greater than median intake (times/day) *
Ratio of vegetable oils (rapeseed oil, sunflower oil, linseed oil, olives) to animal fat (butter, cream, lard) instead of ratio of monounsaturated to saturated fat	Greater than median intake (times/day) *
Red and processed meat: red meat (pork, beef, veal), venison, sausages, ham, liver, entrails, bacon, pate	Lower than median intake (times/day) *

* The reference median of the food frequency consumption in the initial control sample (Table 3).

**Table 3:** Initial control sample characteristics and reference medians of food frequency consumption for the ‘Polish-adapted Mediterranean Diet’ score (0–8) calculation.

Characteristics	Initial Control Sample	
	% or Mean (95% CI)	Median *
*Sample size*	412	
*Sex*		
Men	53.2	
Women	46.8	
*Age* (years)	58.5 (57.8; 59.2)	
*BMI* (kg/m^2^)	28.2 (27.8; 28.7)	
*Frequency of consumption of food groups ^#^*(times/day)		
Vegetables	1.064 (1.010; 1.117)	1.000
Fruit	0.917 (0.867; 0.967)	1.000
Whole grains	0.767 (0.703; 0.832)	0.671
Fish	0.268 (0.238; 0.297)	0.200
Legumes	0.208 (0.181; 0.235)	0.125
Nuts and seeds	0.281 (0.239; 0.323)	0.100
Ratio of vegetable oils to animal fat	1.745 (1.231; 2.258)	0.500
Red and processed meat	1.519 (1.431; 1.607)	1.342

* reference median of food frequency consumption; ^#^ food frequency consumption was expressed as a times/day after assigning the values for categories of frequencies as follows: ‘never or almost never’ = 0; ‘once a month or less’ = 0.025; ‘several times a month’ = 0.1; ‘several times a week’ = 0.571; ‘daily’ = 1; ‘several times a day’ = 2. 95% CI—95% confidence interval.

**Table 4:** Cancer and control sample characteristics (%).

Variable	Cancer-Control Sample	Cancer Sample	Control Sample	*p*-Value
*Sample Size*	560	280	280	
*Sex*				
Men	50.0	50.0	50.0	
Women	50.0	50.0	50.0	
*Age* (years *)	60.9 (7.2)	61.1 (8.0)	60.7 (6.3)	0.4483
*BMI* (kg/m^2^ *) ^a^	27.3 (4.6)	27.0 (5.1)	27.5 (4.1)	0.2006
*Place of residence*				
village	28.8	32.9 ^a^	24.6 ^a^	
town (<20,000 inhabitants)	22.3	23.9	20.7	
town (20–100,000 inhabitants)	20.0	19.3	20.7	0.0304
city (>100,000 inhabitants)	28.9	23.9 ^b^	33.9 ^b^	
*Education level*				
primary	18.6	27.5 ^a^	9.6 ^a^	
secondary	59.3	59.6	58.9	<0.0001
higher	22.1	12.9 ^b^	31.4 ^b^	
*Economic situation*				
below the average	19.5	22.9 ^a^	16.1 ^a^	
average	67.5	66.1	68.9	0.0766
above average	13.0	11.1	15.0	
*Socioeconomic status* ^b^				
low	32.5	41.1 ^a^	23.9 ^a^	
average	16.8	15.4	18.2	<0.0001
high	50.7	43.6 ^b^	57.9 ^b^	
*Physical activity at work* ^c^				
low	51.3	60.4 ^a^	42.1 ^a^	
moderate	33.6	25.7 ^b^	41.4 ^b^	<0.0001
high	15.2	13.9	16.4	
*Physical activity in leisure time* ^d^				
low	28.0	30.7	25.4	
moderate	58.9	60.0	57.9	0.0226
high	13.0	9.3 ^a^	16.8 ^a^	
*Overall physical activity* ^e^				
low	51.8	61.4 ^a^	42.1 ^a^	
moderate	43.6	34.3 ^b^	52.9 ^b^	<0.0001
high	4.6	4.3	5.0	
*Smokers* ^f^	73.4	80.0	66.8	0.0004
*Current smokers*	30.0	32.5	27.5	0.1967
*Former smokers*	71.6	79.3	63.9	<0.0001
*Abuse of alcohol* ^g^	20.5	22.9	18.2	0.1739
‘Polish-aMED’ score (points) *	4.3 (1.9)	4.0 (1.9)	4.6 (1.8)	0.0002
low (0–2)	20.2	26.8 ^a^	13.6 ^a^	
average (3*–*5)	49.6	48.6	50.7	0.0001
high (6–8)	30.2	24.6 ^b^	35.7 ^b^	

‘Polish-aMED’—‘Polish-adapted Mediterranean Diet’ (range of points: 0–8); ^a^ BMI was calculated using measured weight and height; ^b^ was calculated on the basis of place of residence, educational level and declared economic situation (description in the Materials and Methods section); ^c^ physical activity at work: “low”—more than 70% of working time spent sedentary or retired, “moderate”—approx. 50% of working time spent sedentary and 50% of working time spent in an active manner, “high”—approx. 70% of working time spent in an active manner or physical work related to great exertion [39]; ^d^ physical activity in leisure time: “low”—sedentary for most of the time, watching TV, reading books, walking 1–2 h per week, “moderate”—walking, bike riding, gymnastics, gardening, light physical activity performed 2–3 h per week, “high”—bike riding, jogging, gardening, sport activities involving physical exertion performed more than 3 h weekly [39]; ^e^ after combining data based on declared physical activity at work and physical activity in leisure time (Table S3) [40]; ^f^ current or former smokers; ^g^ at least of 1 bottle (0.5 L) of beer or 2 glasses of wine (300 mL) or 2 drinks (300 mL) or 2 glasses of vodka (60 mL) consumption per day [6]; %—sample percentage; * mean and standard deviation (SD); *p*-value—level of significance assessed by chi^2^ test (categorical variables) or Kruskal–Wallis’ test (continuous variables); a-a, b-b—statistically significant differences between the pairs of cancer and control sample, *p* < 0.05.

**Table 5:** Sample percentage (%) and odds ratios (ORs (95% CI)) of breast or lung cancer by adherence to the dietary patterns (*n* = 560).

Dietary Patterns	Tertiles/Levels	Sample Size	%	*p*-Value	Control	Breast or Lung Cancer					
					OR	OR_crude_	95% CI	*p*-Value	OR_adj_	95% CI	*p*-Value
‘Polish-aMED’	low (0–2 points; ref.)	113	66.4		Ref.	Ref.			Ref.		
	average (3–5 points)	278	48.9	<0.001	1.00	0.49	0.31; 0.77	<0.01	0.49	0.30; 0.80	<0.01
	high (6–8 points)	169	40.8		1.00	0.35	0.21; 0.58	<0.0001	0.37	0.21; 0.64	<0.001
‘Prudent’	bottom (ref.)	186	58.6		Ref.	Ref.			Ref.		
	middle	188	44.7	<0.05	1.00	0.57	0.38; 0.86	<0.01	0.67	0.43; 1.05	ns
	upper	186	46.8		1.00	0.62	0.41; 0.94	<0.05	0.73	0.45; 1.67	ns
‘Non-healthy’	bottom (ref.)	187	43.3		Ref.	Ref.			Ref.		
	middle	187	47.6	<0.01	1.00	1.19	0.79; 1.79	ns	0.98	0.64; 1.52	ns
	upper	186	59.1		1.00	1.89	1.25; 2.86	<0.01	1.65	1.05; 2.59	<0.05
‘Dressings and sweetened-low-fat dairy’	bottom (ref.)	186	46.2		Ref.	Ref.			Ref.		
	middle	187	56.1	ns	1.00	1.49	0.99; 2.24	ns	1.50	0.98; 2.31	ns
	upper	187	47.6		1.00	1.06	0.70; 1.60	ns	1.04	0.68; 1.60	ns

‘Polish-aMED’—‘Polish-adapted Mediterranean Diet’ (range of points: 0–8); OR_crude_—crude model; OR_adj_—age (years), sex, type of cancer, BMI (kg/m^2^), socioeconomic status (low, average, high), overall physical activity (low, moderate, high), smoking status (non-smoker, smoker) and abuse of alcohol adjusted model; 95% CI—95% confidence interval; *p*-value—the level of significance was assessed by Wald’s or chi^2^ test; ns—statistically insignificant.

## Supplementary Materials

The following are available online at http://www.mdpi.com/2072-6643/10/4/470/s1, Table S1: The mean (95% CI) of the frequency of food consumption by dietary pattern for cancer-control sample (times/day), Table S2: Potential confounders in the pooled analysis of two Polish case-control studies, Table S3: Estimation of overall physical activity levels after combining data based on self-reported physical activity at work and physical activity in leisure time.
