# Supplementary material for: Associations of Mediterranean Diet and a Posteriori Derived Dietary Patterns with Breast and Lung Cancer Risk: A Case-Control Study
Source: Nutrients. 2018 Apr 11;10(4):470. doi: 10.3390/nu10040470 (PMC5946255; doi:10.3390/nu10040470)
Supplement: Supplementary file 1 [file nutrients-10-00470-s001.pdf]

Table S1. The mean (95%CI) of the frequency of food consumption by dietary patterns for cancer-control sample (times/day)

**Food groups[#]**	**Total**	**Dietary Patterns**											
		**‘Prudent’ tertiles**			**‘Non-Healthy’ tertiles**			**‘Dressings and sweetened-low-fat dairy’ tertiles**			**‘Polish-aMED’ levels**		
		bottom	middle	upper	bottom	middle	upper	bottom	middle	upper	low	average	high
Size (n)	560	186	188	186	187	187	186	186	187	187	113	278	169
Sugar, honey and sweets	2.1 (2.0; 2.2)	2.1 (1.9; 2.3)	2.1 (1.9; 2.3)	2.0 (1.8; 2.2)	1.3 (1.2; 1.5)	1.9 (1.7; 2.0)	3.0 (2.8; 3.2)	1.9 (1.8; 2.1)	2.1 (1.9; 2.2)	2.2 (2.0; 2.4)	2.2 (2.0; 2.5)	2.1 (1.9; 2.2)	1.9 (1.7; 2.1)
Red and processed meats	1.5 (1.4; 1.6)	1.3 (1.2; 1.5)	1.5 (1.4; 1.6)	1.6 (1.5; 1.7)	1.0 (0.9; 1.1)	1.5 (1.4; 1.6)	2.0 (1.9; 2.1)	1.5 (1.4; 1.7)	1.4 (1.2; 1.5)	1.5 (1.4; 1.7)	1.5 (1.3; 1.6)	1.6 (1.5; 1.7)	1.2 (1.1; 1.4)
Animal fats	1.2 (1.1; 1.3)	1.0 (0.9; 1.2)	1.2 (1.1; 1.4)	1.3 (1.2; 1.4)	0.7 (0.6; 0.8)	1.1 (1.0; 1.2)	1.7 (1.6; 1.9)	1.9 (1.8; 2.0)	1.1 (1.0; 1.2)	0.5 (0.5; 0.6)	1.2 (1.1; 1.4)	1.3 (1.2; 1.4)	0.9 (0.8;) 1.0
Milk, fermented milk drinks and curd cheese	1.0 (0.9; 1.0)	0.6 (0.5; 0.6)	1.0 (0.9; 1.1)	1.4 (1.3; 1.5)	0.9 (0.9; 1.0)	1.0 (0.9; 1.1)	1.0 (0.9; 1.1)	0.8 (0.7; 0.9)	1.0 (0.9; 1.1)	1.2 (1.1; 1.3)	0.7 (0.6; 0.8)	1.0 (0.9; 1.0)	1.2 (1.1; 1.3)
Refined grains	1.0 (1.0; 1.1)	1.3 (1.1; 1.4)	1.0 (0.9; 1.1)	0.9 (0.7; 1.0)	0.4 (0.4; 0.5)	1.0 (0.9; 1.1)	1.7 (1.6; 1.8)	1.0 (0.9; 1.1)	1.0 (0.9; 1.1)	1.1 (1.0; 1.2)	1.3 (1.2; 1.4)	1.1 (1.0; 1.2)	0.7 (0.6; 0.8)
Vegetables	1.0 (1.0; 1.1)	0.7 (0.6; 0.8)	1.0 (0.9; 1.1)	1.4 (1.3; 1.4)	1.1 (1.0; 1.1)	1.0 (0.9; 1.1)	1.0 (0.9; 1.1)	1.0 (0.9; 1.1)	1.0 (1.0; 1.1)	1.0 (0.9; 1.1)	0.6 (0.5; 0.7)	1.0 (1.0; 1.1)	1.3 (1.2; 1.4)
Fruits	0.9 (0.8; 0.9)	0.6 (0.5; 0.7)	0.9 (0.8; 0.9)	1.2 (1.1; 1.2)	0.9 (0.8; 1.0)	0.8 (0.7; 0.9)	0.9 (0.8; 1.0)	0.9 (0.9; 1.0)	0.8 (0.8; 0.9)	0.9 (0.8; 0.9)	0.5 (0.5; 0.6)	0.9 (0.8; 0.9)	1.1 (1.1; 1.2)
Whole grains	0.7 (0.7; 0.8)	0.3 (0.3; 0.4)	0.7 (0.6; 0.7)	1.1 (1.0; 1.2)	1.0 (0.9; 1.1)	0.7 (0.6; 0.8)	0.4 (0.3; 0.5)	0.8 (0.7; 0.9)	0.7 (0.6; 0.8)	0.7 (0.6; 0.8)	0.3 (0.2; 0.3)	0.7 (0.6; 0.7)	1.1 (1.0; 1.2)
Potatoes	0.6 (0.6; 0.7)	0.7 (0.6; 0.7)	0.6 (0.6; 0.7)	0.6 (0.6; 0.7)	0.4 (0.4; 0.5)	0.7 (0.6; 0.7)	0.8 (0.8; 0.9)	0.6 (0.6; 0.7)	0.6 (0.6; 0.7)	0.7 (0.6; 0.7)	0.7 (0.6; 0.7)	0.7 (0.6; 0.7)	0.6 (0.5; 0.6)
Vegetable oils	0.6 (0.6; 0.6)	0.4 (0.3; 0.4)	0.6 (0.6; 0.7)	0.8 (0.7; 0.8)	0.5 (0.4; 0.5)	0.6 (0.6; 0.7)	0.7 (0.6; 0.7)	0.6 (0.6; 0.7)	0.6 (0.5; 0.6)	0.6 (0.5; 0.6)	0.4 (0.3; 0.5)	0.6 (0.5; 0.6)	0.7 (0.7; 0.8)
Other edible fats	0.5 (0.4; 0.5)	0.5 (0.4; 0.6)	0.5 (0.4; 0.5)	0.4 (0.3; 0.5)	0.3 (0.3; 0.4)	0.5 (0.4; 0.6)	0.5 (0.4; 0.6)	0.1 (0.1; 0.1)	0.2 (0.2; 0.3)	1.1 (1.0; 1.2)	0.4 (0.3; 0.6)	0.5 (0.4; 0.6)	0.4 (0.3; 0.5)
Cheese	0.4 (0.4; 0.4)	0.3 (0.2; 0.3)	0.4 (0.4; 0.5)	0.5 (0.4; 0.6)	0.3 (0.2; 0.3)	0.4 (0.4; 0.4)	0.5 (0.4; 0.5)	0.4 (0.3; 0.4)	0.4 (0.3; 0.4)	0.4 (0.4; 0.5)	0.3 (0.2; 0.4)	0.4 (0.4; 0.5)	0.4 (0.4; 0.5)
White meat	0.4 (0.4; 0.4)	0.3 (0.3; 0.4)	0.4 (0.4; 0.5)	0.5 (0.5; 0.5)	0.3 (0.3; 0.4)	0.4 (0.4; 0.5)	0.5 (0.5; 0.5)	0.4 (0.3; 0.4)	0.4 (0.4; 0.5)	0.5 (0.4; 0.5)	0.4 (0.3; 0.4)	0.4 (0.4; 0.5)	0.4 (0.4; 0.5)
Juices	0.4 (0.4; 0.5)	0.2 (0.2; 0.3)	0.4 (0.4; 0.5)	0.7 (0.6; 0.8)	0.3 (0.3; 0.4)	0.4 (0.3; 0.5)	0.6 (0.5; 0.7)	0.4 (0.3; 0.5)	0.5 (0.4; 0.5)	0.5 (0.4; 0.5)	0.4 (0.3; 0.5)	0.5 (0.4; 0.5)	0.5 (0.4; 0.6)
Eggs	0.3 (0.3; 0.4)	0.2 (0.2; 0.2)	0.3 (0.3; 0.4)	0.5 (0.4; 0.5)	0.3 (0.2; 0.3)	0.3 (0.3; 0.4)	0.4 (0.4; 0.5)	0.4 (0.3; 0.4)	0.3 (0.3; 0.4)	0.3 (0.3; 0.4)	0.3 (0.2; 0.3)	0.3 (0.3; 0.4)	0.4 (0.4; 0.5)
Sweetened milk drinks and flavored homogenized cheese	0.3 (0.3; 0.3)	0.1 (0.1; 0.2)	0.3 (0.3; 0.4)	0.4 (0.3; 0.5)	0.2 (0.1; 0.2)	0.3 (0.2; 0.4)	0.4 (0.3; 0.5)	0.2 (0.1; 0.2)	0.3 (0.2; 0.3)	0.5 (0.4; 0.5)	0.2 (0.2; 0.3)	0.3 (0.3; 0.4)	0.3 (0.2; 0.4)
Fish	0.3 (0.2; 0.3)	0.1 (0.1; 0.1)	0.2 (0.2; 0.3)	0.5 (0.4; 0.5)	0.3 (0.2; 0.3)	0.3 (0.2; 0.3)	0.3 (0.2; 0.3)	0.3 (0.2; 0.3)	0.3 (0.2; 0.3)	0.3 (0.2; 0.3)	0.1 (0.1; 0.1)	0.3 (0.2; 0.3)	0.4 (0.3; 0.5)
Nuts and seeds	0.2 (0.2; 0.3)	0.1 (0.0; 0.1)	0.2 (0.1; 0.2)	0.4 (0.4; 0.5)	0.4 (0.3; 0.4)	0.2 (0.1; 0.2)	0.2 (0.1; 0.2)	0.3 (0.2; 0.4)	0.2 (0.2; 0.3)	0.2 (0.1; 0.2)	0.0 (0.0; 0.0)	0.2 (0.1; 0.2)	0.5 (0.4; 0.5)
Legumes	0.2 (0.2; 0.2)	0.1 (0.1; 0.1)	0.2 (0.1; 0.2)	0.4 (0.3; 0.4)	0.2 (0.2; 0.3)	0.2 (0.1; 0.2)	0.2 (0.2; 0.3)	0.3 (0.2; 0.3)	0.2 (0.1; 0.2)	0.2 (0.1; 0.2)	0.1 (0.1; 0.1)	0.2 (0.2; 0.2)	0.3 (0.3; 0.4)
Cereals	0.1 (0.1; 0.1)	0.0 (0.0; 0.0)	0.1 (0.1; 0.1)	0.2 (0.2; 0.3)	0.1 (0.1; 0.2)	0.1 (0.1; 0.1)	0.1 (0.1; 0.1)	0.1 (0.0; 0.1)	0.1 (0.1; 0.1)	0.2 (0.1; 0.2)	0.0 (0.0; 0.1)	0.1 (0.1; 0.1)	0.2 (0.1; 0.2)
Sweetened beverages and energy drinks	0.1 (0.1; 0.1)	0.1 (0.0; 0.1)	0.1 (0.1; 0.1)	0.1 (0.1; 0.1)	0.0 (0.0; 0.0)	0.0 (0.0; 0.1)	0.2 (0.1; 0.2)	0.1 (0.1; 0.2)	0.1 (0.1; 0.1)	0.1 (0.0; 0.1)	0.1 (0.0; 0.1)	0.1 (0.1; 0.1)	0.1 (0.0; 0.1)

‘Polish-aMED’ – ‘Polish-adapted Mediterranean Diet’ (range of points: 0-8), levels (in points): ‘low’ (0-2), ‘average’ (3-5), ‘high’ (6-8);
#the frequency consumption was expressed as a times/day after assigning the values for categories of frequency consumption as follows: ‘never or almost never’=0; ‘once a month or less’=0.025; ‘several times a month’=0.1; ‘several times a week’=0.571; ‘daily’=1; ‘several times a day’=2;
95%CI – 95% confidence interval.

Table S2. Potential confounders in the pooled analysis of two Polish case-control studies

**Confounders**	**Response categories**		**Scoring**
**Socioeconomic status (SES)** after combining data based on SES’ single factors	low		1
	average		2
	high		3
**SES’ single factors**			
Place of residence	village		1
	town <20,000 inhabitants		2
	town 20,000-100,000 inhabitants		3
	city >100,000 inhabitants		4
Educational level	primary		1
	secondary		2
	higher		3
Economic situation (self-declared)	below the average		1
	average		2
	above average		3
**Physical activity at work** [39]	low	more than 70% of working time spent sedentary or retired	1
	moderate	approx. 50% of working time spent sedentary and 50% of working time spent in an active manner	2
	high	approx. 70% of working time spent in an active manner or physical work related to great exertion	3
**Physical activity in leisure time** [39]	low	sedentary for most of the time, watching TV, reading books, walking 1-2 hours per week	1
	moderate	walking, bike riding, gymnastics, gardening, light physical activity performed 2-3 hours per week	2
	high	bike riding, jogging, gardening, sport activities involving physical exertion performed more than 3 hours weekly	3
**Overall physical activity**	low		1

after combining data based on physical activity at work and physical activity in leisure time (Table S2)	moderate		2
	high		3
**Abuse of alcohol** [6]	no		1
	yes	intake at least 1 bottle (0.5 liters) of beer or 2 glasses of wine (300ml) or 2 drinks (300ml) or 2 glasses of vodka (60ml) per day	2
**Current-smoker**	no		1
	yes		2
**Former-smoker**	no		1
	yes		2
**Smoking status**	non-smoker		1
	smoker	current-smoker or/ and former-smoker	2

Table S3. Estimate the overall physical activity after combining data based on self-reported physical activity at work and physical activity in leisure time [40]

	Physical activity at work			
		**low**	**moderate**	**high**
Physical activity in leisure time	**low**	low	low	moderate
	**moderate**	low	moderate	moderate
	**high**	moderate	moderate	high
